# Dementia in military and veteran populations: a review of risk factors—traumatic brain injury, post-traumatic stress disorder, deployment, and sleep

**DOI:** 10.1186/s40779-021-00346-z

**Published:** 2021-10-13

**Authors:** Zara Raza, Syeda F. Hussain, Suzanne Ftouni, Gershon Spitz, Nick Caplin, Russell G. Foster, Renata S. M. Gomes

**Affiliations:** 1 Research and Innovation, Blind Veterans UK, 12-14 Harcourt Street, London, W1H 4HD UK; 2 BRAVO VICTOR, Research, 12-14 Harcourt Street, London, W1H 4HD UK; 3 Circadian Therapeutics, Oxford, OX1 1BY UK; 4 School of Psychological Sciences, Monash University, 18 Innovation Walk, Clayton Campus, Wellington Road, Melbourne, VIC 3800 Australia; 5 Sleep & Circadian Neuroscience Institute (SCNi), Nuffield Department of Clinical Neurosciences, Sir William Dunn School of Pathology, Oxford Molecular Pathology Institute, University of Oxford, South Parks Road, Oxford, OX1 3RF UK; 6 Northern Hub for Veterans and Military Families Research, Department of Nursing, Midwifery and Health, Faculty of Health and Life Sciences, Northumbria University, Newcastle, NE7 7XA UK

**Keywords:** Dementia, Alzheimer’s disease (AD), Traumatic brain injury (TBI), Post-traumatic stress disorder (PTSD), Military, Veteran, Deployment, Sleep

## Abstract

The military population face a unique set of risk factors that may increase the risk of being diagnosed with dementia. Traumatic brain injury (TBI) and post-traumatic stress disorder (PTSD) have a higher prevalence in this group in comparison to the civilian population. By delving into the individual relationships between TBI and dementia, and PTSD and dementia, we are able to better explore dementia in the military and veteran populations. While there are some inconsistencies in results, the TBI-dementia association has become more widely accepted. Moderate-to-severe TBI has been found to increase the risk of being diagnosed with Alzheimer’s disease. A correlation between PTSD and dementia has been established, however, whether or not it is a causal relationship remains unclear. Factors such as blast, combat and chemical exposure may occur during a deployment, along with TBI and/or PTSD diagnosis, and can impact the risk of dementia. However, there is a lack of literature exploring the direct effects of deployment on dementia risk. Sleep problems have been observed to occur in those following TBI, PTSD and deployment. Poor sleep has been associated with possible dementia risk. Although limited studies have focused on the link between sleep and dementia in military and veteran populations, sleep is a valuable factor to study due to its association and interconnection with other military/veteran factors. This review aims to inform of various risk factors to the cognitive health of military members and veterans: TBI, PTSD, deployment, and sleep.

## Background

It was estimated that in 2020, there were 5.8 million people aged 65 years and over living with the Alzheimer’s sub-type of dementia in the United States (U.S.) [1]. This estimated value is based on American census data from 2010 and a population study that looked at chronic health conditions within this age group [the Chicago Health and Aging Project (CHAP)]. With the number of people in this age group expected to almost double by 2050, the number of dementia cases is also expected to drastically rise [2]. A survey done by the National Center for Veterans Analysis and Statistics in the U.S. found that the median age of male veterans was 65 [3]. A population survey by the UK Ministry of Defence sub-grouped the veteran population in Great Britain according to age and found that those in the 60 and over age bracket comprised 70.5% of the 2.56 million veterans surveyed [4]. This is the age range that is at greatest risk of developing dementia, and this risk is compounded by the fact that veterans face a unique set of risk factors such as traumatic brain injury (TBI) and post-traumatic stress disorder (PTSD).

The purpose of this review is to highlight research on dementia in military and veteran populations and to discuss the future implications of such knowledge. We aim to do so by exploring TBI, PTSD, military deployment, sleep and the respective relationships of these factors with dementia. We also aim to compare military/veteran and civilian studies, due to a lack of military-focused studies in some areas.

With dementia being an umbrella term used to encompass several neurological conditions and symptoms, many studies use this term instead of specifying the types of dementia. Therefore, in this review, ‘dementia’ is used in instances where the literature has not specified the exact form of dementia. Alzheimer’s disease (AD) is the most common form of dementia and this is reflected in the reviewed literature, with frontotemporal dementia (FTD), Parkinson’s disease dementia, dementia with Lewy bodies and vascular dementia also discussed.

## Search strategy and selection criteria

This is a narrative review where search parameters were used. Searches of PubMed and Scopus were performed to obtain the data and articles for this review. The search terms used were ‘dementia’, ‘Alzheimer’s disease’, ‘traumatic brain injury’, ‘TBI’, ‘post-traumatic stress disorder’, ‘PTSD’, ‘military’, ‘veteran’, ‘deployment’, ‘sleep’. Abstracts and articles were reviewed and included only if they met our criteria of discussing dementia (including AD), and if they were discussed within the context of TBI, PTSD, military deployment, and sleep. There was no date limitation to the articles included.

## Association between TBI and dementia

Military and veteran populations are known to comprise of individuals that experience TBI. Understanding the link between TBI and dementia is, therefore, valuable when exploring dementia in these groups. TBI has been considered a potential risk factor for dementia occurring later in life. Numerous studies have established a link between moderate-to-severe TBI and the risk of developing AD—the most common cause of senile dementia in Europe and U.S. [5]. The key neuropathological signs of AD are amyloid β (Aβ)—containing plaques on the neurons of the brain, tangles of phosphorylated tau proteins within neurons, synapse loss, and neuronal loss [6]. Presently, there are no treatments, nor early diagnostic biomarkers for AD [7]. Abnormal accumulation of proteins in the brain is a commonly observed phenomenon in neurodegenerative diseases. In FTD, the characteristic protein is transactive response DNA-binding protein 43 (TDP-43) [8], while α-synuclein is the hallmark protein for Parkinson’s disease [9]. Following a TBI, accumulation of Aβ, p-tau, α-synuclein, and TDP-43 tends to occur. Since the aforementioned neurodegenerative diseases and TBI share the same hallmark characteristic proteins, it has led to many investigations which explore the exact nature of the relationship between TBI and such diseases.

There are a number of factors that may potentially influence the risk of dementia after TBI [10, 11]. These include cognitive reserve, *ApoE* genotype and age at the time of injury. In addition to these, the risk of developing AD may depend on the severity of TBI. One prospective study on World War II Navy veterans found that individuals who suffered from severe TBI in early adulthood were four times more likely to develop AD, while those with moderate TBI were twice as likely compared to controls. However, there was no link found between mild TBI (mTBI) and a greater risk of AD [12]. The relationship between mTBI and dementia, however, has been explored in civilian settings. Lee et al. [13] used the National Health Insurance database to obtain information for their cohort study. In contrast to the earlier study [12], this study concluded that mTBI is an independent risk factor in developing dementia. As previously mentioned, the risk of developing dementia can also be affected by the age at which the TBI occurs. In another cohort study with a follow-up period of 5–7 years, Gardner et al*.* [14] reported that the risk of developing dementia increased by 26% in those who were 55 years or older, in comparison to controls. Those who had sustained a single moderate-to-severe TBI were at increased risk of developing dementia regardless of age, while a mTBI was enough to raise the risk in those that were 65 years or older. This suggests that for patients who are older, a mild injury is enough to increase susceptibility to developing dementia, but in other age groups, only a moderate or severe injury may be potent enough to cause such effect.

A nationwide study using data from the Finnish Care Register for Health Care performed on the civilian working population (18–65 years) in Finland over a period of 30 years found that, those who suffered from moderate-to-severe TBI (diagnosed between 1987 and 2014) were 90% more likely to develop dementia in comparison to those who had mTBI [15]. Another civilian study used data which contained financial health information. Following adjustment for age, sex and year of index healthcare use, as well as comorbidities such as diabetes and stroke, the study found that there was a 1.68 times increased risk of developing dementia amongst those who had TBI [16].

TBIs are often associated with a period of loss of consciousness (LOC). Crane et al*.* [17] found that TBI with LOC (≤ 1 h) is associated with a greater risk of developing Lewy bodies in civilian populations, although they found no association with AD or dementia. Not all brain injuries are followed by a period of LOC yet, there has been speculation about whether or not TBIs without LOC can also raise the risk of dementia. Bazarian et al*.* [18] found evidence that suggests an association between mTBI with LOC and AD, but insufficient evidence for mTBI without LOC. Barnes et al*.* [19] studied this particular association in a large cohort study of 178,779 veterans who were diagnosed with one or more TBIs in the Veterans Health Administration health care system. They found that the risk of dementia was just over two times greater for those with mTBI without LOC [hazard ratio (*HR*) 2.36, 95% CI 2.10–2.66] after adjusting for comorbidities, and even greater for mTBI with LOC (*HR* 2.51, 95% CI 2.29–2.76). These results may reflect differences in mechanisms which differ according to injury severity. However, the reasoning behind the results’ variation of mTBI with and without LOC remains unclear, as there have been no further studies investigating this since then. Understanding the underlying mechanisms of how TBI with and without LOC may increase dementia risk requires further rigorous research.

Data from a study conducted by Johnson et al*.*[20] showed that several years after a single moderate-to-severe TBI, there was a greater density and distribution of tau and Aβ plaque pathology in comparison to age-matched controls. This suggests that a single TBI may result in the development of long-term neurodegenerative diseases of the AD type. The underlying pathogenesis of AD and the cause behind its increased risk of development in relation to TBI remains unclear. AD is characterised by the presence of extracellular senile plaques consisting of Aβ, and intracellular neurofibrillary tangles which are made of phosphorylated tau proteins [21]. Aβ deposition, tau phosphorylation, and microglial activation are some characteristics which are shared by acute TBIs and AD [22, 23]. One of the key effects of TBI is axonal damage or diffuse axonal injury (DAI) [24]. As a result of DAI, normal axonal transport is disturbed which can then lead to aberrant accumulation of amyloid precursor protein (APP) in the injured regions of the axon alongside cleavage facilitating β-site APP-cleaving enzyme (BACE1) and γ-secretase complex, which are responsible for cleaving APP into Aβ. This can result in abnormally rapid production and expression of Aβ which may, in turn, trigger the cascade of events leading to AD[25].

### TBI and early onset dementia

With considerable evidence supporting an association between TBI and dementia, another interesting area to investigate is whether TBIs can induce earlier development of dementia. One study looked at the risk of dementia in 188,764 older U.S. veterans (≥ 55 years). During a follow-up period of 9 years, it was found that of those who had TBI, 16% developed dementia, in comparison to 10% of those without TBI. It was concluded that over the 9 year period the risk of developing dementia increased by 60%, and the age of onset of dementia lessened by 2 years in comparison to veterans without TBI [26]. A civilian study performed in 2016 which relied on the data obtained from the AD Neuroimaging Initiative determined that in those with a history of TBI, the age of dementia onset occurred at (68.2 ± 1.0) years, i.e. 2 years earlier than in those without a history of TBI (70.9 ± 0.2) years [27]. Early onset dementia or young onset dementia refer to dementia that occurs in those under the age of 65 years. Nordström et al*.* [28] investigated whether the risk of young onset dementia increased as a consequence of TBI in a cohort of 811,622 Swedish men who had been conscripted for compulsory military service. The main outcome of this study was that for non-AD forms of dementia, there was a strong association of young onset dementia with TBIs of varying degrees of severity.

A less common type of dementia, FTD, has also been linked to TBI. A study of a U.S. veteran population (a sample of 554 patients) by Kalkonde et al*.* [29], utilising multivariate analysis, found that TBI is an independent risk factor for FTD; the prevalence of FTD was 4.4 times greater in those with TBI compared to those without. There are a number of possible hypotheses for the mechanism behind the development of FTD as a result of TBI. One is that the frontal and temporal lobes are more susceptible to injury due to their proximity to the rough interior surface of the base of the skull [30]. Another reason may be due to significantly lowered levels of progranulin protein (PGRN). This can occur as a result of a TBI through the activation of the microglia in the brain and the subsequent adhesion of PGRN to other peptides facilitated by elastase, a protease released by microglia, thus lowering PGRN levels [31]. PGRN deficiency has been linked to FTD [32].

There are multiple studies which report a link between TBI and increased risk of dementia, however, it is important to note that the relationship is not yet entirely conclusive as there have been studies that show conflicting results. The risk of dementia due to AD is generally associated with moderate or severe TBIs, but the specific risk is still undetermined as most earlier studies relied purely on International Classification of Diseases (ICD) clinical diagnoses, and did not use any of the recently developed biomarkers or neuropathological examinations for confirmation of aetiology [33, 34]. Complications can, therefore, arise due to the varying definitions for TBI, or misdiagnoses. Another limitation of epidemiologic studies for the TBI-AD relationship is that most of these studies used self-reported information to establish a history of TBI which, if obtained from those who already suffer from cognitive impairment, may not be reliable [33]. Other limitations include bias due to the use of case-controlled studies, reverse causation (undiagnosed AD resulting in TBI), misclassification and differing definitions of neurodegenerative diseases, and insufficient adjustment for covariates (age, sex, race, health, and lifestyle variables e.g. drug or alcohol disorders, depression) [35].

### Long term follow-up studies

Presently, there are very few studies with a long follow-up period. This contributes to the lack of conclusiveness surrounding the nature of the relationship between TBI and dementia since there is a greater possibility of misdiagnoses or reverse causality in studies with a short follow-up period. In a recent study, Nordström et al*.* [36] investigated this association using a large civilian population of 3,329,360 Swedish individuals over the age of 50 years over a long study period of up to 50 years. Overall, the results showed that the association between TBI and an increased risk of a dementia diagnosis was strongest in the first years following a TBI and, although the risk decreased over time, risk remained significantly higher more than 30 years later. It was also found that the risk of being diagnosed with dementia was greater for those that suffered from severe or multiple TBIs than in those who suffered from mild head trauma; a conclusion was also found in a previous study [15]. Another recent civilian study [37], which utilises both a large population and a relatively long follow-up period, is based on data from 2,794,852 Danish citizens who were all over the age of 50 years. In agreement with the Nordström study [36], the risk of dementia was greatest immediately after the TBI. In the first 6 months following a TBI, the risk was about 4 times greater than the control group, and TBI remained a significant risk within the first 4 years after the injury. As seen in the Nordstrom study [36], there is a gradual decrease in the *HR* with time, although at more than 14 years there is still a risk of being diagnosed with dementia (*HR* 1.17, 95% CI 1.13–1.21). Even after adjusting for comorbidities, the risk of dementia was 24% greater in those with a history of TBI compared to those without.

The definition of TBI is uncertain when looking at many studies together, especially depending on the severity of the TBI and often, the lack of differentiation between severities. The possibility of individuals having experienced more than one TBI is not addressed in some studies and may have an impact on the risk of developing dementia. Other risk factors such as age, sex, and genetic status, are often not considered and these should all be taken into account when assessing the overall impact of TBI on dementia risk [11].

One study, focusing on self-reported TBI history in 4761 American autopsy individuals, did not discover a significant association between AD neuropathologic changes and dementia [38]. The report recognises the contradiction of this finding with previous research. In comparison to other studies referenced above, which clinically diagnosed dementia based on ICD codes, this study performed neuropathological examinations. Authors suggest that previous studies which have found an association between TBI and dementia, may have misclassified or misdiagnosed dementia in their samples. This study did not assess the effects of single vs. multiple TBI, nor specify the severities of any of the TBI. Furthermore, a lack of significant association does not necessarily mean that there is no effect. This, and other studies which rely on self-reporting, highlight an interesting point regarding effective and accurate TBI screening. It should be emphasised that the self-reported aspect of TBI, as mentioned before, is difficult to confirm and deduce conclusive information from. Self-reporting of TBI in this study [38] is based on a clinician-led interview, but reporting of symptoms may differ according to how data is gathered and the type of symptoms being reported [39]. It may be more prudent to screen for TBI, especially remote screenings, using self-report questionnaires, clinician-led interviews, detailed physician interviews, and reviewing past hospital reports to ensure accuracy [39, 40].

In recent years, the association between TBI and dementia has become more widely accepted. While the more severe injuries have been accepted as a risk factor for dementia, it has been more difficult to establish whether mTBIs are a risk factor too. A recently conducted meta-analysis consisting of twenty-one studies concluded that mTBIs can indeed contribute to an increased risk of dementia. After pooling together the results of twenty-one studies, authors calculated that individuals with a prior mTBI were 1.96 times more likely to develop dementia [41]. Characterising the effects of TBI is relevant and important outside of the sports and military spheres as these are not the only two populations that are subject to such injuries and their possible short- and long-term effects. With this in mind, the ability to diagnose and treat, or even prevent TBI, may be important factors in reducing the risk and impact of dementia within all populations.

## Correlation between PTSD and dementia

PTSD is another disorder often linked to military and veteran populations, recognition of which is needed to better understand dementia prevalence in military and veteran populations. TBI and PTSD are distinctly different disorders, however, they display some overlapping symptoms, e.g. attention or memory deficiencies, irritability, and sleep disturbances. Both disorders are prevalent in military and veteran populations and it may be difficult to distinguish between the two, especially as they can occur simultaneously [42]. Furthermore, research has shown an interesting association between PTSD and TBIs that have been acquired during deployment on brain volume. TBI causes reduction in volume of the bilateral hippocampus and right medial orbitofrontal cortex [43]. Researchers also found that if an individual has had a TBI, the injury itself may influence how PTSD affects the volume of the amygdala. Amygdala volume was seen to be lower in those with PTSD but not deployment TBI (however, this was not the case when corrected for multiple comparisons). This suggests that TBI may impact amygdala volume in the presence of PTSD [43].

Data collected from 2005 to 2015 shows that there was a total of 138,197 diagnoses of PTSD in individuals deployed during Operation Enduring Freedom, Operation Freedom’s Sentinel, Operation Iraqi Freedom, Operation New Dawn and Operation Inherent Resolve [44]. PTSD has been associated with a greater risk of dementia incidence. Yaffe et al*.* [45] did a retrospective cohort study that focused on 181,093 veterans (≥ 55 years of age) enrolled at Department of Veterans Affairs (VA) health care services in the U.S. Taking into account other factors, for example, medical and neuropsychiatric disorders and demographics of the sample studied, 53,155 individuals who were diagnosed with PTSD had nearly double the risk of developing dementia in comparison to those who did not have PTSD. The strongest link was found between PTSD and FTD.

Qureshi et al*.* [46] investigated the association between PTSD and dementia in older U.S. veterans. The database consisted of veterans (65 years or older) who had PTSD or received a Purple Heart (in this case awarded to members who had only experienced psychological trauma). The veterans were matched and compared to members in a control group by age and sex. Accounting for cofounding factors (e.g. number of clinic visits, TBI, alcohol abuse and dependence, coronary artery disease), this clinical investigation established that veterans with PTSD had double the incidence and prevalence of being diagnosed with dementia as those without PTSD. They also concluded that it is unclear as to whether PTSD itself is a risk factor for dementia or whether the two have any common underlying risk factors, e.g. low baseline cognitive reserves. In a more recent study, however, Bhattarai et al*.* [47] identified PTSD as a significant risk factor for dementia in U.S. veterans, finding a moderate association between the two (*OR* 1.63, 95% CI 1.22–2.19).

### Neuroanatomical effects

In terms of neuroanatomical effects, some studies have shown that PTSD results in a smaller hippocampal volume [48–50], though some show contradictory results [51, 52]. Smaller hippocampal volume has also been linked to AD, and the atrophy of the hippocampus is associated with the deposition of tau and TDP-43 [53]. Wang et al*.* [54] suggested that there may be two possible pathways by which PTSD may induce dementia: 1) a direct pathway that may occur through the over-activation of the hypo-pituitary-adrenal (HPA) axis and the adrenergic system, which are part of the psychological stress pathways, and 2) an indirect pathway whereby trauma induces changes to the anatomy of the brain, increasing the vulnerability and likelihood of developing dementia. Earlier-life trauma (multiple or single but long-lasting) can act as a risk factor, resulting in the constant over-activation of the psychological stress pathway leading to later-life AD [55].

A more recent retrospective study [56] examined the risk of dementia in association with PTSD, and the role of antipsychotic use, in 15,612 male Vietnam veterans (55–65 years at baseline; 2001–2002). The results were not statistically significant, leading the authors to conclude that the risk of dementia does not increase in relation to PTSD. They did, however conclude that the use of antipsychotic drugs may contribute to an increased risk of dementia. The authors commented that the results should be interpreted with caution as the study is purely observational and rely on health diagnostic data, determination of disease severity and cognitive function measures [56]. An additional study of 417,172 U.S. veterans, conducted by Mawanda et al*.* [57], also explored the use of psychotropic drugs and dementia diagnosis in U.S. veterans. A stronger positive relationship between PTSD diagnosis and dementia diagnosis was reported in the group using psychotropic medication, in comparison to the group not using the medication. The patients which had been diagnosed with PTSD and were using selective serotonin reuptake inhibitors, novel antidepressants or atypical antipsychotics were at greater risk of being diagnosed with dementia in comparison to those with PTSD and not using these medications. Such findings suggest that the association between the number of psychotropic medication and dementia risk may be reflective of greater PTSD severity, as well as the presence of other psychiatric comorbidities. Additional research is required to further delineate this relationship.

It is important to note that the onset of dementia can greatly exacerbate the effects of PTSD [58]. Research has also shown that on developing AD, those who have previously experienced PTSD may face reoccurrence or worsening of those symptoms, suggesting a bidirectional link between dementia and PTSD [59]. A meta-analysis quantified this association between PTSD and dementia, identifying PTSD as a risk factor, although the authors added that further research is required in order to determine whether there is a causal link or other contributing cofactors [60]. A meta-analysis by Gunak et al.showed that, amongst veterans, there is a *HR* of 1.61 (95% CI 1.46–1.78) [60]. The link drawn between PTSD and dementia lacks conclusiveness, and the nature of the relationship is unclear. However, there is literature to support the notion of PTSD as a possible risk factor for dementia. Further research is required to better understand this relationship and the impact of other cofactors such as PTSD medication on this relationship. Whether treatment for PTSD can increase or decrease the risk of dementia in an individual should be thoroughly investigated. Furthermore, there needs to be a clear clinical confirmation for a PTSD diagnosis and/or TBI and any possible causality requires further research and confirmation. When considering dementia risk, prevention and severity in military and veteran populations, PTSD is an important factor to assess as it has the possibility to greatly impact a patient’s experience with dementia.

## Military deployment and dementia

Military deployment is another informative factor to research when focusing on dementia in military and veteran populations. The difference between deployed and non-deployed individuals in the military is important when trying to understand the specific neurological effects of combat on the brain. Deployment, in the context of this literature review, can be defined as the period between when a military service member travels to a location for a combat mission and the individual’s return home after the mission [61]. The difference between this experience and that of non-deployed individuals is that non-deployed individuals are not involved in non-training physical combat actions. When examining health problems relating to veterans (for example PTSD, TBI, depression, anxiety), most studies do not take into account or specify whether veterans were deployed or non-deployed [62]. This needs to be taken into consideration when assessing the significance of the study results.

### Deployment and memory

Memory problems have been noted in deployed individuals. Chao [63] and Murphy et al*.* [64] highlight the observation of memory problems in Gulf War veterans returning from deployment. In view of such, Chao [63] investigated the impact of self-reported memory problems in deployed individuals on their performance in objective memory measures. The findings showed lower total learning, retention, and delayed recall scores in comparison to deployed veterans who did not have memory complaints. These results can be of use when assessing the impact of deployment on development of dementia, as subjective memory complaints have previously been linked to dementia incidence [65, 66].

Another study conducted by Chao [67] explored the outcome of chemical exposure during deployment on Gulf War veterans’ brain structure and function. There was an inverse association noted between exposure to chemicals that trigger a chemical alarm and visuospatial ability. A higher frequency of hearing chemical alarms was also related to a lower volume in specific areas of the brain (such as the lateral occipital cortex) responsible for visuospatial control. As problems in visuospatial function have been implicated in early stages of AD development [68] and with posterior cortical atrophy being a form of dementia presenting with visual symptoms [69], it would be useful to investigate deployed individuals who have been exposed to chemicals further in future research. A recently published brief report by Martinez et al*.* [70] shown that deployed U.S. veterans who were exposed to agent orange in the Vietnam era conflict were nearly twice as likely to receive a dementia diagnosis than those not exposed.

Acquired central auditory processing disorder (ACAPD) is a disorder associated with military deployment due to multiple possible factors such as blast exposure, TBI, hazardous noise exposure, and ototoxic solvents [71]. Signs of central auditory dysfunction have also been noted in patients with mild probable AD by Gates et al*.* [72]. It is, therefore, worth exploring the role of deployment on the development of dementia in relation to auditory dysfunction that military personnel may acquire when they are deployed. Another study carried out by Gates et al*.* [73], 13 years later, found that those with impaired memory, with or without dementia, performed worse on auditory processing tests. The researchers hypothesise that both memory and central auditory dysfunction occurring in the same individual may be due to a problem in the frontal lobe. They highlight that isolated memory loss often precedes dementia diagnosis. These findings suggest that additional central auditory testing might be important in screening for early dementia diagnosis.

Overall, there is a current lack of literature exploring the direct link between deployment and dementia development, especially due to studies often not differentiating between deployed and non-deployed participants. It is, therefore, valuable to consider the link between deployment and dementia through the lens of TBI and PTSD. Deployed soldiers expose themselves to life-threatening situations and the possibility of severe injury that can lead to disorders such as PTSD. Soldiers with greater exposure to combat are at greater risk of developing PTSD, in comparison to individuals who have a limited exposure [74]. A study performed on 715 twin veterans who served in Southeast Asia presented a nine-fold increase in the occurrence of PTSD in the twin who experienced combat, in comparison to the other twin who was not deployed [75]. Shen et al*.* [76] studied the impact of differing deployment intensities on PTSD diagnosis. Authors found that those deployed to Iraq or Afghanistan had, respectively, a 6.3% and 1.6% higher probability for positive PTSD screening than those who were not deployed. Another study by Shen et al*.* [77] found the length of deployment to have an effect on PTSD risk; a deployment exceeding 180 days increased odds 1.11 to 2.84 times, in comparison to a shorter deployment period. Meziab et al*.* [78] ran a retrospective study on a sample of U.S. prisoners of war, of which 31% had PTSD. The risk of dementia was increased in veterans who were prisoners of war only, or had PTSD only, but the risk of developing dementia was greatest for those who were prisoners of war and had PTSD. Contrarily, Jones et al. [79] found that deployment itself did not play a role in the increase of PTSD risk. However, exposure to combat during deployment increased the risk of PTSD development. It should be noted that PTSD deployment studies often do not take into account signs of PTSD preceding deployment [80] and, therefore, an observation of dementia as a result of PTSD in deployed individuals may not always be due to their experiences during deployment.

### Deployment and TBI

TBI is another aspect to explore when examining the relationship between deployment and dementia. Regasa et al*.* [81] conducted a retrospective study of 119,353 active-duty U.S. military members and noted the risk of TBI diagnosis to increase 8.4-fold in the 4 weeks after returning from deployment, in comparison to before. During Operation Enduring Freedom and Operation Iraqi Freedom, 60% of wounded individuals exposed to combat were injured due to blasts from explosions [82]. A study of soldiers treated at Walter Reed Army Medical Centre conveyed that close to 60% who sustained injuries from an explosion had a TBI [83]. The concern raised by combat exposure and the associated risk of TBI has also led to the implementation of the Post-Deployment Health Assessment from the Department of Defense and the Veterans Health Administration’s TBI screening questionnaire, which assesses all individuals returning from combat for TBI [84]. However, TBI may not always be a direct result of combat exposure; the Armed Forces Health Surveillance Centre presented data showing that falls/miscellaneous and land transport accidents were the main categories related to TBI-related diagnoses within a combined population in combat and noncombat settings [85]. Furthermore, contrasting results in this area have been reported. The cognitive function observed in a group of 60 Operation Enduring Freedom/Operation Iraqi Freedom service members through neuropsychological testing was similar in those exposed to blasts and those who were not [86]. This has also been noted in other studies [87, 88]. Therefore, blast exposure may not have as significant of an impact on the risk of developing dementia as other factors. However, given the small sample sizes in the studies, the lack of association between blast exposure and cognitive function may reflect low statistical power.

Assessing the impact of deployment itself on dementia risk in military and veteran populations is difficult due to the multiple possible interacting factors. There is a large gap in the literature exploring the direct effects of deployment on dementia development, outside of any intermediary diagnosis, and this should be addressed. The studies discussed in this review, however, explore the individual experiences that military members may go through during deployment, such as injury to visual, auditory and neurological pathways, and their respective relationships to dementia, offering useful insight into the topic. There would be value in improving and adding to post-deployment health examinations that assess for memory, visuospatial and auditory function, as well as thorough investigation for TBI and PTSD, to possibly aid in prevention of dementia in later life.

## Dementia and sleep

TBI, PTSD and military deployment are all factors that may be experienced by military and veteran populations. One aspect these factors all share is the possibility of sleep disturbance. Sleep, therefore, is a valuable part of an individual’s lifestyle to explore when considering the development of dementia in these groups.

### Sleep issues in military and veteran populations

Military and veteran populations are known to suffer from sleep-related problems. Luxton et al*.* [89] explored short sleep duration (SSD) in redeployed Operation Iraqi Freedom service members. Seventy-two percent of 2738 participants were found to sleep 6 h or less, qualifying them for experiencing SSD. Those exposed to combat were also more likely to have SSD than those who were not exposed. Sleep duration of less than 6 h was also discussed to be the most noteworthy predictor for PTSD. The study aids in understanding the interconnected nature of deployment, PTSD and sleep, but further investigation into these topics may assist in understanding the link with dementia.

Peterson et al*.* [90] focused on deployed U.S. Air Force participants and found approximately 75% to report worse sleep quality after their deployment, in comparison to their sleep prior. This was a preliminary study that noted sleep efficiency and sleep onset latency of the participants to be outside of the normal thresholds used to classify insomnia. A cross sectional study also reported that of those military personnel who returned from deployment with sleep disturbances, 88.2% were identified to suffer from a clinically significant sleep disorder, with 63.6% meeting the diagnostic criteria for insomnia [91]. Insomnia disorder was also noted in 57.2% of post-9/11 veterans in another study [92]. Hughes et al*.* [93] discussed an integrated theoretical model to better understand insomnia in military and veteran populations. Authors discuss predisposing factors, precipitating factors and perpetuating factors, with military deployment and military retirement being examples of precipitating factors. These three types of factors convey the complex nature of insomnia and how major life events such as military service can initiate symptoms of insomnia in an individual, or exacerbate pre-existing symptoms. Insomnia symptoms are regarded as chronic, persisting many years after military service in some individuals, highlighting the importance of addressing symptoms early on.

### Sleep as a risk factor

The relationship between problems in sleep and dementia has been explored in civilian populations. Lim et al*.* [94] found that an increase in fragmented sleep led to an increased risk of developing AD. Individuals with the greatest noted sleep fragmentation were also 50% more likely to be at risk of developing AD than those with the lowest noted sleep fragmentation. This suggests a possible link between severity of sleep symptoms and higher likelihood of dementia risk. Shi et al*.* [95] did a systematic review and meta-analysis, noting that those who had sleep disturbances had 1.19 times the risk of developing all-cause dementia than those who did not have sleep disturbances. AD and vascular dementia risk were specifically increased in those who suffered from sleep disturbances. Yaffe et al*.* [96] completed a retrospective cohort study using the data of 179,738 male veterans from the Department of VA. They found that male veterans above the age of 55 who experienced sleep disturbances had a 27% increased risk of developing dementia, compared to those without. This study is a rare example of research discussing the link between sleep and dementia in the military and veteran populations and it focuses mostly on older male veterans. There is a lack of literature in this area and future research is recommended to consider the high number of military members and veterans who experience sleep difficulties.

Although the exact mechanisms underlying the link between dementia and sleep are not fully understood, sleep is known to play an important role in brain health restoration and a lack of sleep could impact brain cognitive function. As the brain does not have a lymphatic system, the way in which it removes toxic waste has been referred to as the ‘glymphatic system’, which has been noted to have an enhanced function during sleep [97]. This may be important in AD pathogenesis as any problems in Aβ clearance may be linked to problems in sleep [98]. A rise in systemic inflammation has also been suggested as a possible mechanism for AD development through increased Aβ burden [99].

### TBI and sleep

Despite few studies have directly explored the association between sleep and dementia in military and veteran populations, there is research on TBI and PTSD, and their respective possible links with sleep. By delving into these relationships, we can try to better understand how much of an impact sleep may have on dementia risk in those with military or veteran backgrounds. One TBI cohort study found 46% of participants with TBI also had a sleep disorder, with obstructive sleep apnoea (OSA) noted in 23% of the group [100]. Insomnia is also known to be a common problem among those with TBI, with one study of 452 individuals with TBI reporting up to 50% of people having insomnia symptoms [101]. Collen et al*.* [102] discussed similar results, with around half of 116 participants with mild-to-moderate TBI presenting with insomnia symptoms. A total of 97.4% of participants in this study reported sleep problems, highlighting the importance of addressing sleep problems in TBI patients. The severity of sleep disturbances and the incidence of insomnia have also been found to increase with experience of multiple TBIs [103]. One contributing factor to consider is that, in those with TBI, melatonin production is significantly lower than in controls [104, 105]. Melatonin is a hormone that is synthesised at night and is involved in regulating the sleep–wake cycle, thus, a reduction in melatonin production may alter sleep–wake cycles. Alongside the impact of the TBI itself, the resulting sleep difficulties following a TBI may additionally contribute to the development of dementia. Dementia is multifactorial and looking at the intermediate symptoms between TBI and dementia may help to establish the link better. The increase in dementia risk following a TBI may not, however, be due to sleep disorders themselves, but the treatments associated with the sleep disorders. Chiu et al*.* [106] focused their research on the use of hypnotics to treat insomnia in TBI patients. They found the risk of dementia to increase in those with TBI and insomnia taking hypnotics, in comparison to no increase in risk observed in TBI patients with insomnia who were not taking hypnotics. Once again, this may be reflective of greater TBI severity and associated comorbidities. The use of hypnotics increased the risk of developing dementia in the population-based study, but experiencing insomnia alone did not. Thus, all factors that may contribute to dementia development are important to explore, including treatment of associated conditions.

### PTSD and sleep

The incidence of sleep disturbances in individuals with PTSD is known to be high, with sleep disturbances possibly implicated in the early stages of PTSD, as well as the severity of PTSD [107]. One meta-analysis of 20 polysomnographic studies found greater stage 1 sleep and lower slow wave sleep in individuals with PTSD than those without [108]. Rapid eye movement (REM) density was also observed to be higher in the PTSD groups, suggesting a possible change in REM function in these patients. Ohayon et al*.* [109] found that 39.6% of participants with PTSD met the Diagnostic and Statistical Manual (DSM) diagnostic standards for insomnia, in comparison to 6.5% of participants without PTSD. One review hypothesised the possibility of associations between sleep, PTSD and dementia through hippocampal damage [110]. Hippocampal volume has been observed to be decreased in those with PTSD [111]. Interestingly, chronic sleep deprivation has also been associated with a reduction of hippocampal volume which may contribute to the development of cognitive disorders and psychiatric diseases [112]. However, further research is needed to determine the extent and effect of hippocampal changes in associating sleep and dementia in PTSD patients. Neylan et al. [113] conducted a study on Vietnam veterans and found a higher percentage of veterans reporting difficulty falling asleep, difficulty maintaining sleep and experiencing nightmares in the group with PTSD than in the group without PTSD, or the civilian group. The possible sleep problems that may arise in patients following PTSD diagnosis could contribute to the overall dementia risk of the individual. It is useful to consider the involvement of sleep when looking at the link between PTSD and dementia.

By exploring the impact of sleep on the development of dementia in military and veteran populations, through the lens of TBI, PTSD and deployment, we are able to look more holistically at dementia patients who are part of these communities. Although there is research still to be done in order to understand the interaction of these factors better, the knowledge we already have about sleep could be implemented to help these patients. Problems in sleep have been implicated in the time following deployment, TBI and PTSD. Therefore, addressing sleep problems in deployed individuals or TBI and PTSD patients at an early stage would be valuable, and may play a part in understanding dementia risk, treatment, and prevention.

## Other risk factors

It is recognised that other factors such as depression, diabetes, hypertension, and other traditional risk factors may also play an important part in the development of dementia in military populations. Yet, this paper focused on reviewing associations between the following risk factors: TBI, PTSD, deployment, and sleep.

## Conclusion

**Fig. 1:**
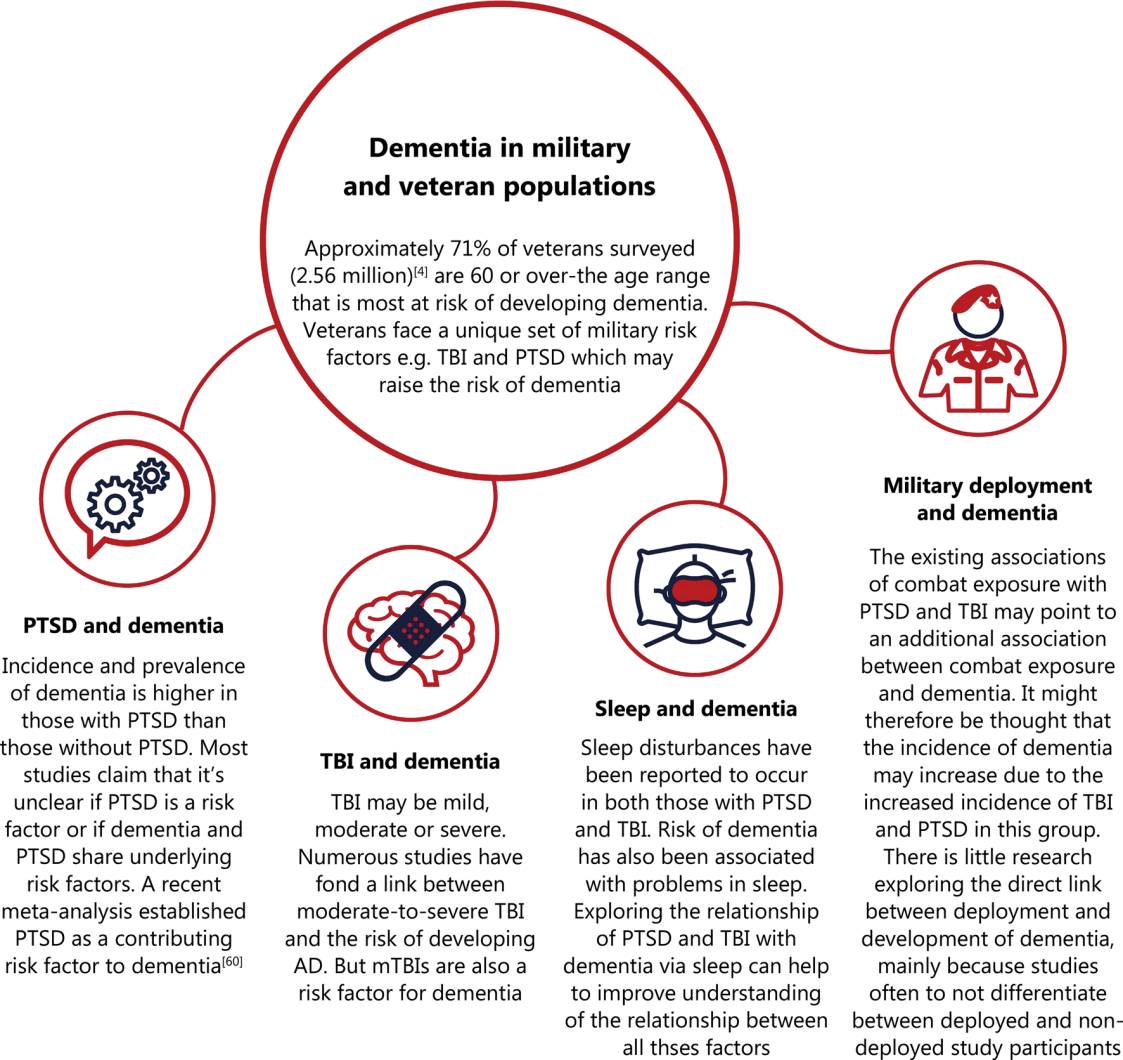
Summary of risk factors and associations for dementia in military and veteran populations based on the literature discussion to hand. AD Alzheimer’s disease, TBI traumatic brain injury, PTSD post-traumatic stress disorder, mTBI mild TBI

Military deployment, TBI, PTSD and poor sleep might all contribute to dementia development in military and veteran populations, with differing measures of impact (Fig. 1). This is due to the complex nature of dementia and the complexities of military and veteran experiences. The link between TBI and dementia appears to be the strongest from the current literature reviewed, with moderate-to-severe TBI increasing the risk of dementia development. PTSD, however, has also been associated with dementia risk, but further research to confirm the nature of this association is required. Deployment is usually noted to precede TBI and PTSD diagnosis in military and veteran populations, however, diagnoses can also occur independent of military deployment. Deployment itself may also affect memory directly, outside of TBI and PTSD diagnoses. Problems in sleep have been observed following military deployment, TBI and PTSD, however these can also occur independently, and can impact dementia development on their own. The exact order and nature of the interactions between TBI, PTSD, deployment and sleep are difficult to establish, however, it is possible that these factors may all simultaneously influence each other to increase risk of dementia development. Further research exploring the direct effects of deployment on dementia is recommended, along with research specifically focusing on the link between sleep and dementia in military and veteran populations. By recognising TBI, PTSD, deployment and poor sleep as possible factors for dementia development in military and veteran populations, we can try to better understand the complex nature of dementia and aid in the prevention, treatment and prognosis of dementia patients.

## Data Availability

Not applicable.

## Abbreviations

ACAPD: Acquired central auditory processing disorder
AD: Alzheimer’s disease
APP: Amyloid precursor protein
Aβ: Amyloid β
BACE: β-Site APP-cleaving enzyme
CHAP: Chicago Health and Aging Project
DAI: Diffuse axonal injury
DSM: Diagnostic and Statistical Manual
FTD: Frontotemporal dementia
HPA: Hypo-pituitary-adrenal
*HR*: Hazard ratio
ICD: International Classification of Diseases
LOC: Loss of consciousness
mTBI: Mild TBI
OSA: Obstructive sleep apnoea
PGRN: Progranulin protein
REM: Rapid eye movement
PTSD: Post-traumatic stress disorder
SSD: Short sleep duration
TBI: Traumatic brain injury
TDP-43: DNA-binding protein 43
U.S.: United States
VA: Veterans Affairs
